# Daptomycin-induced eosinophilic pneumonia - a systematic review

**DOI:** 10.1186/s13756-016-0158-8

**Published:** 2016-12-12

**Authors:** Priyasha Uppal, Kerry L. LaPlante, Melissa M. Gaitanis, Matthew D. Jankowich, Kristina E. Ward

**Affiliations:** 1 Providence Veterans Affairs Medical Center, Providence, RI USA; 2 Department of Pharmacy Practice, University of Rhode Island, 7 Greenhouse Rd, Suite 295 J, Kingston, RI 02881 USA; 3 Warren Alpert Medical School of Brown University, Providence, RI USA

**Keywords:** Daptomycin, Eosinophilia, Pneumonia

## Abstract

**Purpose:**

Eosinophilic pneumonia comprises a group of lung diseases in which eosinophils appear in increased numbers in the lungs and sometimes in the bloodstream. Several case reports link daptomycin use to this phenomenon.

**Summary:**

We performed a systematic literature review to identify cases of eosinophilic pneumonia associated with daptomycin use. Relevant studies were identified by searching Pubmed/Medline, EMBASE, Google Scholar, Cochrane Database of Systematic Reviews, and Clin-Alert from inception to May 2016, and manual searches of reference lists. All case reports that include information regarding patient age, indication, clinical and objective findings, treatment and outcome were evaluated. Abstracts from conference proceedings as well as case reports not in English were excluded. Descriptive statistics were used to analyze the data. Thirty-five patient-cases were included in the final analysis. Patients most likely to be identified with daptomycin-induced eosinophilic pneumonia were male (83%) and elderly (mean age 65.4 ± 15 years). The dose for daptomycin ranged from 4 to 10 mg/kg/day, but included a large number of patients with renal dysfunction. The average duration of daptomycin therapy upon onset of EP symptoms was 2.8 ± 1.6 weeks. Majority of patients presented with dyspnea (94%), fever (57%) and were also found to have peripheral eosinophilia (77%) and infiltrates/opacities of CT/CXR (86%). Symptom improvement was seen after daptomycin discontinuation (24 h to 1 week). The majority of patients were also prescribed treatment with corticosteroids (66%).

**Conclusion:**

Clinicians should be aware of daptomycin-induced eosinophilic pneumonia and its symptoms along with its presentation and treatment.

## Background

Eosinophilic pneumonia is a rare, but serious respiratory syndrome that occurs when eosinophils accumulate in the lungs [1, 2]. It has been associated with several medications and chemicals, with antibiotics and nonsteroidal anti-inflammatory drugs among the most common [3, 4].

The pathophysiology of acute eosinophilic pneumonia is thought to be caused by detection of an antigen by alveolar macrophages which leads to recruitment of T-helper 2 lymphocytes and subsequent release of interleukin 5. Interleukin 5 promotes eosinophil production and migration to the lung. Additionally, eotaxin (a potent eosinophil chemoattractant) production by alveolar macrophages, pulmonary endothelial cells, airway smooth muscle cells, and alveolar epithelial cells leads to further accumulation of eosinophils in the lungs [5].

Daptomycin is a cyclic lipopeptide antibiotic derived from the fermentation of *Streptomyces roseosporus*. Daptomycin has activity against Gram-positive organisms including methicillin-resistant *Staphylococcus aureus* (MRSA) and vancomycin-resistant enterococci (VRE) [6]. In 2007, pulmonary eosinophilia was added to the “Adverse Reactions, Post-Marketing Experience” section of the product label for daptomycin [2]. A review of the literature and the US FDA Adverse Event Reporting System database in 2012 revealed 7 definite, 13 probable, and 38 possible cases of daptomycin-induced eosinophilic pneumonia [1]. While the mechanism of daptomycin’s pulmonary toxicity is not known, the drug undergoes conformational change through interaction with calcium which allows binding to the cytoplasmic membrane, increased membrane permeability, and intracellular ion escape [7]. The antibacterial activity of daptomycin is decreased because of its binding to pulmonary surfactant. Some have speculated two potential mechanisms for daptomycin’s pulmonary toxicity: 1) chronic daptomycin administration results in drug accumulation near the epithelial alveolar surface causing epithelial injury and pneumonia and 2) the daptomycin-surfactant interaction could alter lipid integrity which may stimulate an inflammatory response [3, 7, 8].

Per the FDA guidance, eosinophilic pneumonia is attributed to daptomycin when the following criteria are met: 1) concurrent exposure to daptomycin, 2) fever, 3) dyspnea with increased oxygen requirement or requiring mechanical ventilation, 4) new infiltrates on chest x-ray or computed tomography (CT) scan, 5) bronchoalveolar lavage (BAL) with > 25% eosinophils, and 6) clinical improvement following daptomycin withdrawal [2]. Solomon and Schwartz [4] have also developed criteria for drug- or toxin-induced eosinophilic pneumonia that is similar and includes 1) presence of simple, acute, or chronic eosinophilic pneumonia by diagnostic criteria which includes excess of eosinophils either on lung biopsy or BAL (usually ≥25%) in the setting of parenchymal infiltrates 2) presence of a potential candidate drug or toxin in an appropriate time frame 3) no other cause of eosinophilic pneumonia such as fungal or parasitic infection 4) clinical improvement after cessation of the drug or toxin, and 5) recurrence of eosinophilic pneumonia with re-challenge to the drug or toxin. However, re-challenge is often not recommended as it can be dangerous [4, 5].

Although the mechanism of daptomycin-induced eosinophilic pneumonia is unknown, some have speculated that daptomycin may bind to human surfactant and accumulate in the alveolar space causing injury to the epithelium with resulting inflammation [7, 8]. The purpose of this review is to systematically evaluate the published literature describing daptomycin-induced eosinophilic pneumonia.

## Methods

All relevant cases and studies were identified by systematically searching of the PubMed, EMBASE, Google Scholar, Cochrane Database of Systematic Reviews, and Clin-Alert databases by two reviewers from inception through May 2016. The truncated terms “daptomycin”, “eosinophil*”, and “pneumon*” were searched in each database. All case reports that included information regarding patient age, indication, clinical and objective findings, treatment, and outcome were evaluated. Reports not published in English were excluded as well as abstracts from conference proceedings. Descriptive analysis was used to present pooled demographic information and other data where applicable.

## Results

No clinical or observational trials assess daptomycin-induced eosinophilic pneumonia; only case reports and case series are published. In 2012, Kim et al. identified 7 definite, 13 probable, and 38 possible cases of daptomycin-induced eosinophilic pneumonia via review of literature and FDA Adverse Event Reporting System Reports (AERS) as defined in Table 1 [1]. Details regarding the 38 possible cases reported through AERS were not described. Of the 20 cases that were identified as definite or probable, 9 have been published in the literature, the remaining 11 were summarized by Kim [1, 3, 9–13]. We also identified 39 additional cases of eosinophilic pneumonia attributed to daptomycin for a total of 59 cases described in the literature [7, 14–24]. Of those, 21 were excluded from this systematic review because they were abstract presentations at international meetings and did not go through the peer review publishing process [25–41]. Another three were excluded because they were not published in English [42–44]. Currently available data on a total of 35 cases of daptomycin-induced eosinophilic pneumonia is summarized in Table 2.

**Table 1:** Criteria for inclusion as definite, probably, possible, and unlikely cases of daptomycin-induced eosinophilic pneumonia [1, 2]

Definite	Probable	Possible	Unlikely
Concurrent exposure to daptomycin	Concurrent exposure to daptomycin	Concurrent exposure to daptomycin	All other cases that did not meet criteria
Dyspnea with increased oxygen requirement or requiring mechanical ventilation	Dyspnea with increased oxygen requirement or requiring mechanical ventilation	New infiltrates on CXR or CT	
New infiltrates on CXR or CT	New infiltrates on chest x-ray or CT	Clinical improvement following daptomycin withdrawal OR the patient died	
BAL with > 25% eosinophils	BAL with ≤ 25% eosinophils OR peripheral eosinophilia		
Clinical improvement following daptomycin withdrawal	Clinical improvement following daptomycin withdrawal		
Fever			

Adapted from references 1 and 2

Abbreviation: *BAL* bronchoalveolar lavage; *CT* computed tomography; *CXR* chest x-ray

**Table 2:** Summary of 35 cases of presumed daptomycin-induced eosinophilic pneumonia

Case	Age/Sex	Indication	Dose (mg/kg/day)	DAP Duration (wks)	Clinical Findings	Objective Findings	Treatment	Outcome
Kim [1] (2012)	63/F	MSSA spinal osteomyelitis	6	3	• Fever • Cough, hypoxemia	• BAL = 60–70% • Peripheral eosinophilia • Elevated CPK	• DAP d/c • Corticosteroids	Recovered
	64/M	Osteomyelitis with bacteremia	5.7	4	• Fever • Dyspnea, hypoxia	• BAL = 44% • Peripheral eosinophilia • Pulmonary infiltrates	• DAP d/c	Recovered
	79/M	Endocarditis	6	6	• Fever, cough, night sweats • Dyspnea requiring MV	• BAL = 9–13% • Peripheral eosinophilia • CT = ground glass opacities • Lung biopsy = eosinophilic pneumonitis	• DAP d/c • Corticosteroids	Improved
	26/M	MRSA bacteremia	7.35	1.4	• Dyspnea requiring MV	• BAL not performed • Peripheral eosinophilia • Pulmonary infiltrates • Eosinophils in tracheal aspirate	• DAP d/c	Improved
	43/M	MRSA osteomyelitis	6	1–2	• Pleuritic pain • Hypoxia requiring O_2_	• BAL not performed • Peripheral eosinophilia • CT = bilateral infiltrates	• DAP d/c • Given NSAIDs, meperidine	• Improved • Residual infiltrates on CT s/p 4 wks
	66/M	MSSA bacteremia	6	1	• Dyspnea requiring O_2_ • Hematemesis	• BAL with eosinophils (not quantified) • Peripheral eosinophilia	• DAP d/c • Corticosteroids	Recovered
	71/M	MRSA diabetic foot infection	4	7.7	• Dyspnea requiring O_2_	• Peripheral eosinophilia • Elevated CRP • Elevated ESR • CT = bilateral interstitial opacities	• DAP d/c	Improved
	77/F	Bacteremia (enterococcal)	5	1	• Dyspnea requiring O_2_	• Peripheral eosinophilia • CXR = pneumonitis	• DAP d/c • Corticosteroids	Improved
	67/M	MRSA endocarditis	6	4.3	• Dyspnea requiring MV	• BAL = 9% • Peripheral eosinophilia • CT = bilateral pulmonary infiltrates	• DAP d/c • Corticosteroids	Improved
	73/M	Prosthetic joint infection	5	3.7	• Fever • Dyspnea requiring MV	• Peripheral eosinophilia • CT = bilateral ground glass appearance	• DAP d/c • Corticosteroids	Recovered
	81/F	MRSA paraspinal abscess	6	1.6	• Dyspnea requiring MV	• BAL = 2% (s/p corticosteroid)^a^ • CXR = bilateral mid-lung infiltrates	• DAP d/c • Corticosteroids	Improved
Cobb [6] (2007)	84/M	Infection of left knee prosthesis	4	4	• Decreased appetite • Weight loss • Fatigue • Weakness	• Elevated ESR • CT with infiltrates • Lung biopsy = eosinophilic pneumonia	• DAP d/c	• Improved within 2 weeks
Hayes [7] (2007)	60/M	MSSA endocarditis	NR	2	• Fever, rigors, diaphoresis • Required MV	• BAL 16% initially • BAL 26% after rechallenge • CRP elevated	• DAP d/c – then re-challenged • DAP d/c plus corticosteroids	• Rechallenge failed within 4 h • Improved within 24 h after DAP d/c
Kakish [8] (2008)	65/M	MRSA vertebral osteomyelitis, epidural abscess	6	2	• Low-grade fever • Dyspnea requiring MV	• BAL = 33% • Peripheral eosinophilia • Lung biopsy revealed eosinophils	• DAP d/c • Corticosteroids	• Improved within 72 h • Normal CT at 3 months
Shinde [9] (2009)	54/M	Complicated inguinal hernia repair	NR	2	• Low grade fever, cough • Hypoxemia requiring MV	• Peripheral eosinophilia • CT = bilateral airspace, peripheral predominance, small bilateral effusions • Lung biopsy = many eosinophils	• DAP d/c • Corticosteroids	• Improved within 24 h • Normal CT at 4 weeks
Lal [10] (2010)	82/M	Prosthetic joint infection	NR	3	• Fever • Hypoxia requiring O_2_	• BAL = 14% • Peripheral eosinophilia • CT = patchy bilateral infiltrates	• DAP d/c • Corticosteroids	• Recovered after 5 days • Recurrent symptoms • Low dose steroids required
	87/M	Prosthetic knee infection	NR	4	• Dyspnea, dry cough requiring O_2_ • Malaise, chills, anorexia, fever	• BAL = 40% • Peripheral eosinophilia • CT = bilateral patchy pulmonary infiltrates	• DAP d/c • Corticosteroids	• Recurrence s/p steroid taper • Low dose steroids for 2 years
Miller [11] (2010)	60/M	MSSA prosthetic hip infection	6	2	• Cough, fever • Hypoxia requiring O_2_	• BAL = 81% after rechallenge • Peripheral eosinophilia • CT = bilateral scattered ground-glass opacities • Lung biopsy = acute fibrinous and organizing pneumonia, reactive alveolar and interstitial epithelial changes	• DAP d/c • Rechallenged, DAP d/c • Corticosteroids	• Improved within 48 h • Rechallenge failed within 24 h
	60/M	MRSA osteomyelitis, septic arthritis	6	2	• Non-productive cough, dyspnea • Low-grade fevers, chills	• Peripheral eosinophilia • CT = patchy peripheral nodular/ground-glass	• DAP d/c	• Resolution within 96 h • Recurrence with re-challenge at 5 months
	83/M	Diskitis of lumbar spine	6	4	• Progressive dyspnea, • Cough, pleuritic chest pain	• BAL = 13% • Peripheral eosinophilia • CT = diffuse ground-glass, reticular opacities • Lung biopsy = acute organizing pneumonia, eosinophilia, chronic inflammation, fibro-inflammatory changes	• DAP d/c • Corticosteroids	Improved within 6 days
Kalogeropoulous [12] (2011)	78/M	Endocarditis	8	1.4	• Fever, chills, diaphoresis, • Hypoxemia requiring O_2_	• BAL = 27.5% • Peripheral eosinophilia • Elevated ESR • Elevated CRP • CT = patchy consolidation, ground-glass opacities, bilateral pleural effusions	• DAP d/c	Resolution within 24 h
Rether [13] (2011)	69/M	Spondylo-discitis with lumbar epidural and bilateral psoas abscesses	6	3	• Fever • Dyspnea requiring O_2_	• BAL = 30% • Elevated CRP • CXR = extensive patchy infiltrates in RLL and entire left lung	• DAP d/c • Corticosteroids	Improved within 24 h
Patel [14] (2014)	61/F	Osteomyelitis	NR	1	• Dry cough • Dyspnea requiring MV	• BAL = 30% • Peripheral eosinophilia • CT = bilateral pleural effusion, diffuse bilateral patchy infiltrate	• DAP d/c • Corticosteroids	Improved within 72 h
Phillips [15] 2014)	48/M	Osteomyelitis	6	3	• Fever • Dyspnea requiring MV	• BAL = 17% • Peripheral eosinophilia • CXR = patchy bilateral airspace opacities	• DAP d/c • Corticosteroids	• Improved
	28/M	Osteomyelitis	6	4	• Dyspnea requiring MV • Chest pain, light-headedness	• BAL = 74% • Peripheral eosinophilia • CT = diffuse multi-lobar infiltrates	• DAP d/c • Corticosteroids	Resolution within 1 week
Yamamoto [16] (2014)	82/M	MRSA bacteremia	10	2	• Low grade fever • Hypoxia	• CT = bilateral ground glass opacities • Sputum negative for eosinophils	• DAP d/c	Improved
Yusuf [17] (2014)	64/M	Prosthetic joint infection	10	4	• Fever	• BAL = 47% • Peripheral eosinophilia • Elevated CRP • CT = diffuse bilateral ground-glass opacities	• DAP d/c	Improved within 24 h
	61/M	Prosthetic joint infection	10	2	• Fever • Dyspnea requiring MV	• BAL = 3% • Peripheral eosinophilia • Elevated CRP • CT = ground-glass consolidation, bilateral pleural effusion	• DAP d/c	Improved within 24 h
Chiu [18] (2015)	77/M	Osteomyelitis	6	6	• Pleuritic chest pain • Cough, dyspnea requiring O_2_	• BAL = 18% • Elevated CRP • CXR = diffuse bilateral airspace disease	• DAP was d/c 1 day before symptoms • Corticosteroids	Improved within 60 h
	74/F	Infected hip reconstruction	6	1^b^	• Fever • Dyspnea requiring O_2_	• CXR – bilateral airspace disease	• DAP d/c • Corticosteroids	Improved within 24 h
Hagiya [19] (2015)	34/M	Endocarditis	10	1	• Cough with mild hypoxemia	• Peripheral eosinophilia • Elevated CRP • CT = consolidation in peripheral field of right upper lobe	• DAP d/c	Resolved within 6 weeks
Hatipoglu [20] (2015)	67/F	MRSA diabetic foot ulcer	NR^c^	3.3	• Cough, dyspnea requiring BPAP • Fever, fatigue, decreased appetite	• Peripheral eosinophilia • Elevated CRP • CT = right lobe infiltration	• DAP d/c • Inhaled corticosteroids	Improved within 72 h
Roux [21] (2015)	67/M	MSSA prosthetic hip infection	6	2.4	• Dry cough, hypoxemia	• BAL = 10% • Peripheral eosinophilia • CT = diffuse alveolar and interstitial opacities	• DAP d/c • Corticosteroids	Improved within 96 h
Wojtaszczyk [22] (2015)	76/M	Septic arthritis and pacemaker vegetation	NR	2	• Dyspnea requiring O_2_, cough • Fever, fatigue	• BAL = 58% • Elevated CRP • CT = bilateral ground glass opacity, patchy consolidation	• DAP d/c • Corticosteroids	Resolved within 72 h
Akcaer [23] (2016)	60/M	MSSA post-amputation abscess	5	3.4	• Tachypnea, hypoxia requiring O_2_	• Peripheral eosinophilia • Elevated CRP • Elevated ESR • HRCT = right pleural effusion, bilateral tree-in-bud pattern, bilateral scattered ground-glass opacities	• DAP d/c	Resolved within 72 h

KEY: *BAL* bronchoalveolar lavage, *BPAP* bilevel positive airway, *CPK* creatine phosphokinase, *CRP* C-reactive protein, *CT* computed tomography scan, *CXR* chest x-ray, *DAP* daptomycin, *d/c* discontinued, *ESR* erythrocyte sedimentation rate, *F* female, *HRCT* high resolution computed tomography, *M* male, *MRSA* methicillin resistant *Staphylococcus aureus*, *MSSA* methicillin susceptible *Staphylococcus aureus, MV* mechanical ventilation, *NR* not reported, *NSAID* nonsteroidal anti-inflammatory drug*, O*_*2*_ oxygen, *RLL* right lower lobe; *s/p* status post, *wks* weeks

^a^ = DAP given for 1 week, then held for 2 weeks, and restarted. Symptom onset in 72 h after restarting, ^b^ = not included in analysis, ^c^ = DAP 500 mg/day given (dose mg/kg unknown)

Analysis of the 35 cases shows eosinophilic pneumonia resulting from daptomycin use is most likely to be reported in males with a mean age of 65.4 ± 15 years and a mean length of therapy of 2.8 ± 1.6 weeks at symptom onset. The most common indication for daptomycin use was osteomyelitis and/or diabetic foot infection closely followed by prosthetic joint infection. Daptomycin dose ranged from 4 to 10 mg/kg/day depending on renal function; therefore, the adverse effect does not appear to be dose dependent, but time dependent exposure. The most common symptoms of eosinophilic pneumonia included fever and dyspnea often requiring either oxygen supplementation or mechanical ventilation. Other clinical findings included malaise, elevated ESR (4/35 cases), or elevated C-reactive protein (11/35 cases). Peripheral eosinophilia was also present in approximately 77% (27/35 cases) of patients. Many cases also had computed tomography scans or chest x-rays which revealed opacities (12/35 cases) and bilateral infiltrates (13/35 cases). Symptom improvement was seen within 24 h through one week after daptomycin discontinuation. The majority of patients were also prescribed treatment with corticosteroids (23/35 cases); however, all patients were reported to recover (See Table 3).

**Table 3:** Compilation of available data on 35 cases of daptomycin-induced eosinophilic pneumonia

Sex, n (%)	
Male	29 (83)
Female	6 (17)
Age(years), mean ± SD	65.4 ± 15
Daptomycin indication, n (%)	
Osteomyelitis/diabetic foot infection	11 (31)
Prosthetic joint infection	9 (26)
Endocarditis	5 (14)
Bacteremia	4 (11)
Abscess	3 (9)
Other	3 (9)
Daptomycin dose (mg/kg/day), mean ± SD	6.4 ± 1.6
Treatment duration at symptom onset (weeks), mean ± SD	2.8 ± 1.6
Clinical findings, n (%)	
Dyspnea	33 (94)
Fever	20 (57)
Cough	13 (37)
Requiring oxygen	15 (43)
Requiring mechanical ventilation	12 (34)
Infiltrates/opacities of CT/CXR, n (%)	30 (86)
BAL eosinophils %, mean ± SD	32 ± 22.4
Peripheral eosinophilia, n (%)	27 (77)
Lung biopsy consistent with AEP, n (%)	6 (17)
Treatment, n (%)	
Daptomycin discontinued only	12 (34)
Daptomycin discontinued plus corticosteroid	23 (66)

*AEP* acute eosinophilic pneumonia, *BAL* bronchoalveolar lavage, *CT* computed tomography, *CXR* chest x-ray, *SD* standard deviation

## Discussion

While the criteria developed by Solomon and Schwartz differ from the FDA guidance, they are largely similar in that an offending agent (here, daptomycin) must be present, >25% eosinophils are present, and that clinical improvement is seen after discontinuation of the drug. The FDA guidance also includes some measures of symptomatology such as fever and dyspnea [2, 4].

Overall, dyspnea was the most common documented symptom associated with eosinophilic pneumonia followed by the presence of either pulmonary infiltrates or opacities on chest x-ray or CT. A total of 10 cases specifically mentioned the characteristic finding of ground glass opacities on CT. A potential limitation of this review is that some of the daptomycin-induced eosinophilic pneumonia used lung biopsy in place of BAL as part of the diagnostic criteria which is not part of the FDA guidance, but is included in the Solomon and Schwartz criteria. In addition, since some patients had BAL < 25% eosinophils but lung biopsy revealed eosinophilia [1, 13], a 25% cut off may be too strict in certain situations.

Corticosteroids are believed to be beneficial at halting clinical manifestations of daptomycin-induced eosinophilic pneumonia and were used in the majority of reported cases. Steroids exert action through eosinophilic apoptosis and through accelerating intracellular signaling involved in eosinophil death [45]. No dose or length of corticosteroid treatment is established in guidelines for eosinophilic pneumonia; however, a commonly employed regimen is intravenous methylprednisolone 60–125 mg every 6 h, with conversion to prednisone 40–60 mg oral daily and taper over 2–6 weeks. Use of a 2- or 4-week course appears to have similar time to resolution of clinical symptoms and radiological abnormalities [46].

## Conclusion

As use of daptomycin continues to increase, it is important for clinicians to recognize and appropriately manage daptomycin-induced eosinophilic pneumonia. Although symptoms may resolve upon discontinuation of daptomycin, use of corticosteroid may be beneficial for recovery. Further research is needed to determine the exact mechanism of daptomycin-induced eosinophilic pneumonia and identify the optimal treatment course.

## Abbreviations

AERS: Aderverse Event Reporting System
BAL: Bronchoalveolar lavage
CT: Computed tomography
CXR: Chest x-ray
EP: Eosinophilic pneumonia
ESR: Erythrocyte sedimentation rate
FDA: Food and Drug Administration
MRSA: Methicillin resistant Staphylococcus aureus
VRE: Vancomycin resistant enterococcus

## References

[1] Kim PW, Sorbello AF, Wassel RT (2012). Eosinophilic pneumonia in patients treated with daptomycin: review of the literature and US FDA adverse event reporting system records. Drug Saf.

[2] US Food and Drug Administration. FDA drug safety communication: eosinophilic pneumonia associated with the use of Cubicin (daptomycin). http://www.fda.gov/Drugs/DrugSafety/PostmarketDrugSafetyInformationforPatientsandProviders/ucm220273.htm. (Accessed 30 Nov 2016).

[3] Allen JN (2004). Drug-induced eosinophilic lung disease. Clin Chest Med.

[4] Cubicin (daptomycin) package insert. Lexington, MA: Cubist Pharmaceuticals; 2006 May.

[5] Silverman JA, Mortin LI, Vanpraagh AD (2005). Inhibition of daptomycin by pulmonary surfactant: in vitro modeling and clinical impact. J Infect Dis.

[6] Cobb E, Kimbrough RC, Nugent KM, Phy MP (2007). Organizing pneumonia and pulmonary eosinophilic infiltration associated with daptomycin. Ann Pharmacother.

[7] Hayes D, Anstead MI, Kuhn RJ (2007). Eosinophilic pneumonia induced by daptomycin. J Infect.

[8] Kakish E, Wiesner AM, Winstead PS, Bensadoun ES (2008). Acute respiratory failure due to daptomycin induced eosinophilic pneumonia. Respir Med.

[9] Shinde A, Seifi A, DelRe S (2009). Daptomycin-induced pulmonary infiltrates with eosinophiiia. J Infect.

[10] Lal Y, Assimacopoulos AP (2010). Two cases of daptomycin induced eosinophilic pneumonia and chronic pneumonitis. Clin Infect Dis.

[11] Miller BA, Gray A, Leblanc TW, Sexton DJ (2010). Acute eosinophilic pneumonia secondary to daptomycin: a report of three cases. Clin Infect Dis.

[12] Kalogeropoulos AS, Tsiodras S, Loverdos D (2011). Eosinophilic pneumonia associated with daptomycin: a case report and a review of the literature. J Med Case Rep.

[13] Rether C, Conen A, Grossenbacher M, Albrich WC (2011). A rare cause of pulmonary infiltrates one should be aware of: a case of daptomycin-induced acute eosinophilic pneumonia. Infection.

[14] Patel JJ, Atony A, Herrera M, Lipchik RJ (2014). Daptomycin-induced acute eosinophilic pneumonia. WMJ.

[15] Phillips J, Cardile AP, Oatterson TF, Lewis JS (2013). Daptomycin-induced acute eosinophilic pneumonia: Analysis of the current data and illustrative case reports. Scand J Infect Dis.

[16] Yamamoto K, Hayakawa K, Ohmagari N (2014). Daptomycin-induced pneumonitis in a patient with chronic obstructive pulmonary disease (COPD). Intern Med.

[17] Yusuf E, Perrottet N, Orasch C (2014). Daptomycin-associated eosinophilic pneumonia in Two patients with prosthetic joint infection. Surg Infect.

[18] Chiu SY, Faust AC, Dand HM (2015). Daptomycin-induced eosinophilic pneumonia treated with intravenous corticosteroids. J Pharm Pract.

[19] Hagiya H, Hasegawa K, Asano K (2015). Myopathy and eosinophilic pneumonia coincidentally induced by treatment with daptomycin. Intern Med.

[20] Hatipoglu M, Memis A, Turhan V et al. Possible daptomycin-induced acute eosinophilic pneumonia in a patient with diabetic foot infection. Int J Antimicrob Agents. 2016;47:414–5. doi:10.1016/j.ijantimicag.2016.02.014 10.1016/j.ijantimicag.2016.02.014 27089828

[21] Roux S, Ferry T, Chidiac C, Valour F (2015). Daptomycin-induced eosinophilic pneumonia. Int J Infect Dis.

[22] Wojtaszczyk A, Jankowich M (2015). Dyspnea on daptomycin: eosinophilic pneumonia. RI Med J.

[23] Akcaer M, Karakas A, Tok D et al. Eosinophilic pneumonia: daptomycin-induced lung complication. Med Mal Infect. 2016 http://dx.doi.org/10.1016/j.medmal.2016.01.006 10.1016/j.medmal.2016.01.006 26965755

[24] Prahl JD, Tripp MS, Stafford CM. Organizing pneumonia and pneumothorax associated with daptomycin use. Chest. 2010; 138 (4) Suppl: 77A1-2. American College of Chest Physicians Annual Meeting October 30 - November 4, 2010; Vancouver, British Columbia, Canada. doi: 10.1378/chest.9330

[25] Brixey AG, Willers ED, Ferrel P, Light RW. Daptomycin-induced eosinophilic pneumonia. Am J Resp Crit Care Med. 2011; 183(1): A3876. American Thoracic Society 2011 International Conference, May 13–18, 2011; Denver, CO doi: 10.1164/ajrccm-conference.2011.183.1_MeetingAbstracts.A3876

[26] Chow L, Gorga J, Zein J. Development of new pulmonary infiltrates in a patient treated with daptomyinc. Chest. 2011:140(4_Meeting Abstracts):159A American College of Chest Physicians Annual Meeting October 22–26, 2011; Honolulu, Hawaii. doi:10.1378/chest.1117499.

[27] Gaines J, Mills A. Daptomycin-induced eosinophilic pneumonia. J Hosp Med. 2011;6 (4 Suppl 2): S181-182. Society of Hospital Medicine 2011 Abstracts Research, Innovations, Clinical Vignettes Competition, Hospital Medicine, May 10–13, 2011; Grapevine, Texas. doi: 10.1002/jhm.920 10.1002/jhm.920 21491585

[28] O’Brien C. Acute eosinophilic pneumonia secondary to daptomycin: A potentially fatal adverse drug reaction. J Hosp Med. 2011;6 (4 Suppl 2): S224-225. Society of Hospital Medicine 2011 Abstracts Research, Innovations, Clinical Vignettes Competition, Hospital Medicine, May 10–13, 2011; Grapevine, Texas. doi: 10.1002/jhm.920

[29] Mehta P, Wong W, Ramalingam S et al. Chest. 2012:142(4_Meeting Abstracts):1042A. American College of Chest Physicians Annual Meeting October 20–25, 2012; Atlanta, Georgia. doi: 10.1378/chest.1380652.

[30] Safadi B, Carlos W, Bosslet G. Daptomycin-induced eosinophilic pneumonia. Chest. 2012:142(4_Meeting Abstracts):485A. American College of Chest Physicians Annual Meeting October 20–25, 2012; Atlanta, Georgia. doi:10.1378/chest.1388121.

[31] Tolle LB, Hyzy RC. A 73-year-old man with acute eosinophilic pneumonia owing to daptomycin. Am J Resp Crit Care Med. 2012;185:A4620. American Thoracic Society 2012 International Conference, May 18–23, 2012; San Francisco, California. doi: 10.1164/ajrccm-conference.2012.185.1_MeetingAbstracts.A4620

[32] Edukulla J, Choudhary S, Glick A, DeSouza D. Eosinophilic pneumonia, rare but fatal side effect of daptomycin: A case report. J Gen Intern Med. 2013;28 (Suppl 1):S324. 36th Annual Meeting of the Society of General Internal Medicine April 24–27, 2013; Denver, Colorado. doi: 10.1007/s11606-013-2436-y.

[33] Azam M, Asghar S, Aggen D et al. Acute eosinophilic pneumonia: A rare side effect of daptomycin. Chest. 2014; 146 (4_Meeting Abstracts):179A. American College of Chest Physicians Annual Meeting October 25–30, 2014; Austin, Texas. doi:10.1378/chest.1989619.

[34] Folkard C, Munoz R. Daptomycin-induced eosinophilic pneumonia. J Gen Intern Med. 2014;29 (Suppl 1): S341-342. 37th Annual Meeting of the Society of General Internal Medicine, April 23–26, 2014; San Diego, California. doi: 10.1007/s11606-014-2834-9.

[35] Neyra K, Rahman A, Gupta, V, Cohen Z, Nafees. Acute eosinophilic pneumonia due to exposure to daptomycin leading to ventilator-dependent respiratory failure. Chest. 2014;148 (4_Meeting Abstracts):635A. American College of Chest Physicians Annual Meeting October 25–28, 2015; Montreal, Quebec, Canada. doi: 10.1378/chest.2247139

[36] Rajagopal A, Mintz E, Reese L. Daptomycin-induced eosinophilic pneumonia without Peripheral eosinophilia. Chest. 2014;145(3_MeetingAbstracts):127A. CHEST World Congress 2014, March 21–24, 2014; Madrid, Spain. doi: 10.1378/chest.1775453

[37] Kuchlean D, Ie S, Foroozesh M. Daptomycin a pulmonary foe. Am J Respir Crit Care Med. 2015;191:A1661. American Thoracic Society 2015 International Conference, May 15–20, 2015; Denver, Colorado. doi: 10.1164/ajrccm-conference.2015.191.1_MeetingAbstracts.A1661

[38] Hilal T, Khosravi M. Daptomycin-induced acute eosinophilic pneumonia. Am J Respir Crit Care Med. 2015;191:A1533. American Thoracic Society 2015 International Conference, May 15–20, 2015; Denver, Colorado. doi: 10.1164/ajrccm-conference.2015.191.1_MeetingAbstracts.A1533

[39] Goyal P, Breen MJ, Hountras P et al. Daptomycin-induced acute eosinophilic pneumonia: Case report and review of literature. Am J Respir Crit Care Med. 2016; 193:A1658. American Thoracic Society 2016 International Conference, May 13–18, 2016; San Francisco, California. doi: 10.1164/ajrccm conference.2016.193.1_MeetingAbstracts.A1658.

[40] Hirai J, Kinjo T, Hagihara M et al. Eosinophilic pneumonia caused by daptomycin: Five case reports and review of the literature. Am J Respir Crit Care Med. 2016;193:A1586. American Thoracic Society 2016 International Conference, May 13–18, 2016; San Francisco, California. doi: 10.1164/ajrccm-conference.2016.193.1_MeetingAbstracts.A1586

[41] Komur S, Ulu A, Kurtaran B et al. [Sudden respiratory failure and eosinophilic pneumonia in patients treated with daptomycin: a report of five cases]. *Cukurova Med J*. 2016; 41): 396–399. doi:10.17826/cutf.208261. Turkish

[42] Montenegro O, Del Campo R, Del Rio JJ, Ambrós Checa A. [Acute eosinophilic pneumonia secondary to daptomycin]. Enferm Infecc Microbiol Clin. 2015 Oct 31. doi: 10.1016/j.eimc.2015.08.010. Spanish. 10.1016/j.eimc.2015.08.010 26530225

[43] Søndergaard TS, Schumacher H, Norup K (2010). Possible daptomycin-induced organizing pneumonia. Ugeskr Laeger.

[44] Solomon J, Schwarz M (2006). Drug-, toxin-, and radiation therapy induced eosinophilic pneumonia. Semin Respir Crit Care Med.

[45] Druilhe A, Létuvé S, Pretolani M (2003). Glucocorticoid-induced apoptosis in human eosinophils: mechanism of action. Apoptosis.

[46] Rhee CK, Min KH, Yim NY (2013). Clinical characteristics and corticosteroid treatment of acute eosinophilic pneumonia. Eur Respir J.

